# A common TLR1 polymorphism is associated with higher parasitaemia in a Southeast Asian population with *Plasmodium falciparum* malaria

**DOI:** 10.1186/s12936-015-1071-y

**Published:** 2016-01-06

**Authors:** William O. Hahn, Susanna Harju-Baker, Laura K. Erdman, Srivicha Krudsood, Kevin C. Kain, Mark M. Wurfel, Wayne C. Liles

**Affiliations:** Division of Allergy and Infectious Diseases, Department of Medicine, Harborview Medical Center, University of Washington, Box 359640, Seattle, WA USA; Division of Pulmonary Critical Care Medicine, Department of Medicine, Harborview Medical Center, University of Washington, Seattle, WA USA; Tropical Disease Unit, Department of Medicine, University Health Network-Toronto General Hospital, Toronto, Ontario Canada; SAR Laboratories, Sandra Rotman Centre for Global Health, University of Toronto, Toronto, Ontario Canada; Clinical Malaria Research Unit, Faculty of Tropical Medicine, Mahidol University, Bangkok, Thailand

**Keywords:** Plasmodium falciparum, Single nucleotide polymorphisms, Toll-like receptor 1, Toll-like receptor 6, Sepsis, Parasitaemia

## Abstract

**Background:**

The factors leading to poor outcomes in malaria infection are incompletely understood. Common genetic variation exists in the human genes for Toll like receptors (TLRs) that alter host responses to pathogen-associated molecular patterns. Genetic variation in *TLR1* and *TLR6* could alter the risk of development of complicated malaria and ability of the host to control the parasite burden during acute *Plasmodium falciparum* infection.

**Methods:**

Five single nucleotide polymorphisms in *TLR1* and *TLR6* in 432 patients with clinical *P. falciparum* monoinfection acquired on the Thai-Myanmar border were genotyped. Using logistic regression, associations with the development of complicated malaria and the percentage of infected erythrocytes (parasitaemia) on the day of presentation to clinical care (day zero) were tested.

**Results:**

Genotypes carrying the T (major) allele of *TLR1* rs5743551—an allele associated with improved outcomes in sepsis—were associated with higher parasitaemia measured on day zero (p = 0.03).

**Discussion:**

Since malaria exerts strong genetic pressure on the human genome, protection from parasitaemia associated with *TLR1* rs5743551 may account for the maintenance of an allele associated with poor outcomes in Caucasians with sepsis.

**Conclusion:**

These data suggest that genetic variation in *TLR1* has effects on the host response to *Plasmodium falciparum* malaria in Asian populations. Genotypes from *TLR6* showed no evidence of association with either complicated malaria or parasite burden.

## Background

Malaria is estimated to cause approximately 850,000 deaths per year with most mortality occurring before the age of 5 years [1]. Since death occurs prior to the age of reproduction, the malaria parasite is thought to exert strong selective pressure on the human genome. The clearest example is the sickle cell trait where the heterozygote state provides protection against malaria whereas homozygosity for sickle cell trait (sickle cell disease) is uniformly fatal. The high prevalence of several independently arising alleles causing sickle cell trait in different populations implies a strong selective pressure on the human genome [2]. While the immunologic host determinants of susceptibility to malaria remain incompletely understood, family-based longitudinal studies have demonstrated that susceptibility to clinical malaria is partially heritable [3].

The role of innate immunity and, specifically, innate immune responses in host defence against malaria has been reviewed previously [4]. Common genetic variations in TLR genes have been associated with disease susceptibility in humans to a wide variety of pathogens [5]. Polymorphisms in innate sensing pathways have also been implicated in host susceptibility to malaria [6]. There are several components of the malaria parasite that have been implicated as pathogen-associated molecular patterns (PAMPs) recognizable by the innate immune system, including glycosylphosphatidylinositol (GPI) anchors on the surface membranes of infected red blood cells (RBCs) and DNA-haemozoin complexes within the cytoplasm [7, 8]. GPI initiated signals are thought occur via TLR1/2 or TLR2/TLR-6 heterodimers and DNA-hemozoin complexes via TLR9 [8–10]. TLR1 is part of a complex of TLR receptors that includes TLR1, TLR6, and TLR10, and is thought to have arisen via a gene duplication event [11].

Since *Plasmodium* is a well-described cause of the sepsis syndrome, the possible contribution of *TLR *polymorphisms to clinical outcomes in malaria also has potential explanatory power [12, 13]. In a cohort of patients with sepsis and septic shock from an area with no ongoing malaria transmission, it has been shown that common variation in the gene for TLR1, the G (minor) allele of *TLR1*_7202A/G_ (rs5743551) is associated with a large increase in the severity of organ dysfunction and an increased risk of death [14]. Others have observed similar biologic effects of this hyperfunctional variant and have shown this allele to be associated with increased risk for clinical tuberculosis, leprosy and frequency of leprosy reversal reactions [15, 16]. The minor allele of *TLR1*_7202A/G_ has been also been associated with increased Interleukin-6 (IL-6) production and decreased regulatory T cell (CD4+, CD25+) mediated suppression of proliferation utilizing in vitro culture systems [17]. The *TLR1*_7202A/G_ (minor allele) is a non-coding SNP that sits within the 5′ upstream region of the TLR1 gene and, thus, cannot have a direct effect on TLR1 protein function. It has been previously demonstrated, however, that the rs5743551 SNP is associated with increased secretion of soluble mediators of inflammation in response to stimulation with Pam3CysSerLys4 (Pam3CSK4), a specific ligand for TLR1/TLR2 heterodimers [14]. One potential explanation for the observed hyper responsiveness is that *TLR1* rs5743551 is in LD with several coding polymorphisms that strongly increase cell surface expression of TLR1.

These data suggest that polymorphisms in *TLR1 *have functional consequences in immunologic signalling and influence the host response to a broad range of microbial pathogens. Given the potential role of TLR1 in inflammatory responses to malaria, it was hypothesized that the presence of the minor *TLR1*_*A7202G*_ allele would be associated with malaria severity and the magnitude of parasitaemia.

## Methods

### Malaria patients

Patients (age >13 years) were enrolled from a population presenting with febrile illness to clinical sites for Hospital for Tropical Disease (Mahidol University) on the Thai-Myanmar border found to be infected with *Plasmodium falciparum* malaria. Study recruitment was from 2000–2004, a period in which malaria was endemic. Patients were excluded if they had another plausible medical explanation for fever (e.g., lobar pneumonia). The age of 13 was selected as subjects were originally recruited to participate in a previously reported clinical trial [18]. Both thick and thin smears were performed on whole blood specimens from all patients. Enrolment occurred on the basis of unblinded smear microscopy, but this was confirmed with PCR (as previously described) [19]. Patients with mixed infections were excluded. Patients were initially classified into uncomplicated malaria (UM), severe malaria or cerebral malaria on the basis of WHO criteria [20].

An expert microscopist blinded to other diagnostic testing confirmed the presence of *P. falciparum* peripheral blood smear after enrolment and performed quantitation of the parasite. Species level infection was also confirmed with PCR as previously described [21]. Parasite density was determined by counting the number of parasites per 200 white blood cells for thick blood films or per 1000 RBCs for thin blood films.

The institutional review board of the Faculty of Tropical Medicine, Mahidol University approved this study. Verbal and written explanations were provided in the patient’s’ native language, and informed consent was obtained from all patients or their legal guardians prior to specimen collection.

### Genotyping

*TLR1* (rs5743551, rs5743614, rs5743618) and *TLR6* (rs3775073, rs5743808) in the malaria cases were genotyped using Taqman-based real-time polymerase chain reaction (RT-PCR) as previously described [22]. Primer-probe sets were obtained from the Applied Biosystems repository (*TLR1*_−*7202A/G*_) or synthesized (*TLR1*_1517G/A_, forward primer: AGCAGCCTTTCTGTATTGATCATTGA, reverse primer: CATCTTCTGGCAGCTCTGGAA, probe: CACCCATC[G/A]GCTGAT; *TLR1*_1804G/T_, forward primer: GGTGTTGGCTGTGACTGTGA, reverse primer: GCACACCATCCTGAGATACCA, probe: CCTCTGCA[G/T]CTACTT) and run according to the recommended guidelines on an ABI Prism 7900HT (Applied Biosystems, Inc., Foster City, CA, USA). 2 % of the samples were replicated for quality control.

#### Statistical analysis

The two phenotypes tested for association with *TLR1* and *TLR6* genotypes were:

(1) Complicated malaria disease versus uncomplicated disease, and (2) log_10_-transformed measurements of parasitaemia on day 0 for each subject. Observed genotype frequencies were compared with expected frequencies to test for deviations from Hardy–Weinberg equilibrium as previously described [23]. Inspection of the genotype frequencies by ethnicity revealed that patients that were self-declared “Karen” exhibited genotype frequencies highly divergent from the other major ethnic groups (data not shown). Patients of Karen ethnicity were excluded to avoid confounding due coexistent but unrelated population-specific differences in genotype frequencies and phenotype prevalence. Cambodian and Nepali patients were also excluded due to very low population sizes. To identify associations between *TLR1* and *TLR6* genotypes and the presence of complicated malaria in the Burmese, Mon, and Thai patients, logistic regression was used, assuming an additive effect as previous work demonstrated an intermediate functional phenotype in heterozygotes [14]. To test for an association between *TLR1* and *TLR6* genotypes and day 0 log_10_ transformed parasitaemia, linear regression was used assuming an additive effect. For associations detected in the un-adjusted analyses, multiple linear regression analyses were performed adjusting for age and gender. Finally, to assess whether genotypic effects were consistent across ethnic groups, sensitivity analyses were performed with stratification on the basis of each of the three major ethnic groups (Mon, Burmese, and Thai). Statistical significance was set at p < 0.05. Analyses were performed using Stata 10.0 (Stata Corp., College Station, TX, USA).

## Results

### *TLR1* genotype frequency and susceptibility to complicated malaria

**Fig. 1:**
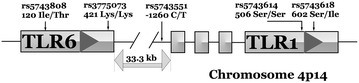
Genetic maps of TLR1 and TLR6. *Diagram* shows the transcriptional orientation, exon/intron structure, intergenic distance and location of genotyped polymorphisms (dbSNP reference number, nucleotide alleles and amino acid alleles for coding variants) for *TLR1* and *TLR6*

Common genetic variants within *TLR1* and *TLR6* are associated with altered innate immune responses to synthetic bacterial lipoproteins [14]. Functional genetic variation within *TLR1* and *TLR6* could therefore be associated with severity of malarial illness and/or the degree of parasitaemia observed in each patient on the day patients were diagnosed with clinical malaria. To test this hypothesis five different single nucleotide polymorphisms (SNPs) were genotyped, three in *TLR1* and two in *TLR6* (Fig. 1), in a group of 435 patients enrolled in northern Thailand with PCR-confirmed infection with *P. falciparum*.

Self-reported race/ethnicity in the studied group revealed the existence of several populations with the majority of patients declaring Thai ancestry (41.8 %) followed by Mon (29.0 %), Karen (16.3 %), Burmese (12.2 %), and, to a much lesser extent Cambodian and Nepali (Tables 1, 2). The patients were young with a mean age of 27 years. In terms of severity of illness, 59 % had UM while 19.8 % had cerebral malaria and 21.2 % had complicated disease. Mean parasitaemia on day 0 was nearly 80,000 parasites/uL and ranged from 13 to over 1 × 10^6^ parasites/uL. As expected, disease severity and parasitaemia were correlated. Mean parasitaemia in patients with cerebral or complicated malaria was roughly an order of magnitude higher than in patients with uncomplicated malaria (p < 0.01) (Table 2). Given the relatively small number of patients with complicated disease and the assumption that TLR responses are likely to be an early event in malaria host defense within the causal pathway for both forms of complicated malaria, it was decided to pool cerebral and complicated malaria for the purposes of analysis. This approach is consistent with natural history studies in African children where severe clinical malaria exists as a series of overlapping syndromes rather than discrete clinical phenotypes [24].

**Table 1:** Demographic characteristics of enrolled patients

N	432
Age, mean (std dev)	27 (11.1)
Ethnicity, n (%)	
Thai	182 (41.8)
Mon	126 (29.0)
Karen	71 (16.3)
Burma	53 (12.2)
Cambodia	2 (0.5)
Nepali	1 (0.2)

**Table 2:** Clinical characteristics of enrolled patients

	N	Age	Sex		D0 Parasitemia (/ul)
		Median (range)	% F	% M	Median (range)
Uncomplicated	257	25 (2–65)	32.3	67.7	7890 (13–422,400)
Severe	92	22 (12–59)	25.3	74.7	71,400 (350–944,240)
Cerebral	86	26 (14–61)	19.4	80.6	95,790 (94–1,188,720)

Consistent with prior reports documenting this divergence using other genetic markers, genotype frequencies for patients of Karen ethnicity were markedly different from other major ethnic groups and are divergent from frequencies observed in reference Asian populations [25, 26]. This was not due to assay variability, as genotyping was successful with over 98 % of all genotypes called with excellent agreement between replicates (>99 %) and all genotype frequencies were found to be consistent with Hardy–Weinberg equilibrium. Given the likelihood that associations between *TLR1* or *TLR6* genotype frequencies and malaria phenotypes would be confounded by major underlying population differences in genetic architecture, analyses for the Karen subjects were performed separately from the other ethnic groups. Patients who self-reported Burmese, Mon, or Thai ethnicity were analysed as a group.

A trend towards an association between the “major” T allele of *TLR1* rs5743551 (associated with less inflammatory responses in other systems) and susceptibility to complicated malaria [OR (CI) = 1.26 (0.93–1.71), p = 0.14, Table 3] was observed. When analysed within each ethnic group, all three groups (Burma, Mon, and Thai) exhibited trends of similar direction and magnitude but none met pre-specified criteria for statistical significance (data not shown). No major trends were observed with any other polymorphism tested nor was there any significant association observed between any genotype and susceptibility to complicated malaria in subjects of Karen ethnicity.

**Table 3:** Genotype associations

Gene	Genotype	Malaria type				Parasitemia (day 0)		
		Uncomplicated	Complicated	OR (CI)	*p*	Mean	β	*p*
*TLR1*	rs5743551							
	CC	59 (64.8)	32 (35.2)	1.26 (0.93–1.71)	0.14	57,224 (91,846)	0.16	*0.042*
	CT	107 (58.5)	76 (41.5)			73,597 (142,004)		
	TT	44 (53.7)	38 (46.3)			106,744 (190,805)		
	rs5743614							
	AA (Ser/Ser)	59 (62.8)	35 (37.2)	1.17 (0.86–1.59)	0.32	57,116 (89,419)	0.14	0.078
	AG (Ser/Ser)	110 (58.5)	78 (41.5)			73,087 (142,004)		
	GG (Ser/Ser)	41 (55.4)	33 (44.6)			111,539 (198,734)		
	rs5743618							
	TT (Ile/Ile)	202 (58.6)	143 (41.5)	1.41 (0.48–4.12)	0.53	79,114 (147,719)	−0.03	0.91
	GT (Ser/Ile)	7 (50.0)	7 (50.0)			55,513 (73,399)		
	GG (Ser/Ser)	0	0			–		
*TLR6*	rs3775073							
	TT (Lys/Lys)	55 (57.9)	40 (42.1)	0.89 (0.66–1.18)	0.42	87,461 (143,708)	0.07	0.35
	TC (Lys/Lys)	106 (63.1)	62 (36.9)			81,825 (167,173)		
	CC (Lys/Lys)	49 (52.1)	45 (47.9)			61,273 (99,746)		
	rs5743808							
	AA (Ile/Ile)	158 (58.5)	112 (41.5)	0.96 (0.62–1.48)	0.85	79,744 (150,930)	−0.04	0.74
	GA (Thr/Ile)	47 (57.3)	35 (42.7)			73,177 (132,271)		
	GG (Thr/Thr)	5 (71.4)	2 (28.6)			67,254 (77,658)		

### *TLR1*_−7202A/G_ is associated with elevated malaria parasitaemia

**Fig. 2:**
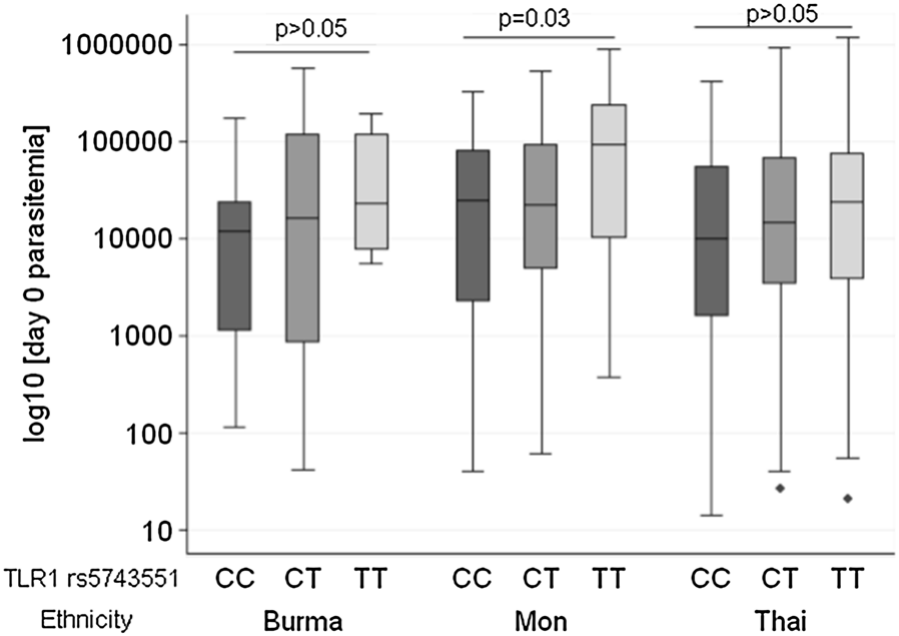
Parasitaemia by TLR1 rs5743551 in major ethnic groups. *Box plots* represent the median (*mid-box line*), intra-quartile range (*upper* and *lower box* margins), *upper* and *lower* values within ± 1.5 × intra-quartile range (*upper* and *lower* *error bars*), and outliers (*blue filled circles*) for parasitaemia measured on day 0 of enrollment by TLR1 rs5743551 genotype

Associations between malaria parasitaemia measured on day 0 of enrollment and *TLR1* and *TLR6* genotypes were also tested. Table 3 shows an association between increasing copy number of the T-allele (major allele) of *TLR1* rs5743551 and higher levels of parasitaemia. A nearly two-fold increase in mean parasitaemia in subjects carrying the “major” TT genotype relative to those carrying the “minor” GG genotype (Table 3) was observed. Notably, when analysed by ethnicity, all three major ethnic groups demonstrated a similar trend with the major allele with the strongest effect observed in subjects of Mon ethnicity (p = 0.03) (Fig. 2). A similar trend was observed with *TLR1* rs5743614 although this did not meet statistical significance. No other genotypes demonstrated an association with parasitaemia. In addition, no genotypes were found to associate with day 0 parasitaemia in subjects of Karen ethnicity.

## Discussion

Early host innate immune inflammatory responses are likely to play an important role in determining the clinical course after infection with *P. falciparum*. It was determined that *TLR1* polymorphisms previously demonstrated to be associated with functional changes in immunologic signaling were associated with different levels of host control of *P. falciparum* in patients with clinical malaria. In the studied patient population, the GG (minor) allele of rs5743551 was associated with a parasite density roughly half of that of patients with the TT (major) allele. The G (minor) allele has been associated with increased inflammatory cytokine production in response to bacterial lipopeptides that are recognized by TLR1/2 heterodimers [14]. The direction of this association with the G (minor) allele in the studied population of patients with clinical malaria, however, is opposite the direction in sepsis patients where the presence of the T (major) allele was associated with improved clinical outcomes. Furthermore, the density of the pathogen is different in patients with the G (minor) allele with respect to malaria and sepsis, as carriers of the minor G allele were more likely to have blood cultures with bacterial growth (a proxy for degree of bacterial burden) compared to carriers of the major T allele whereas the minor G allele was associated with fewer parasites per millilitre of blood. This implies that the influence of innate signaling molecules on the ability of the host to control an infectious process is more than simply the degree of inflammatory mediators produced. The G (minor) allele may have persisted in the population because early inflammation may be important to control parasite replication during acute malaria infection, whereas hyperresponsiveness to other PAMPS is detrimental in other disease states (e.g. bacterial sepsis).

Investigations into the role of TLR signalling in malaria infection using murine models have revealed conflicting results. Both TLR1 and TLR6 signal via forming heterodimers with TLR2 [9, 10] All TLRs except for TLR3 signal via the adaptor protein MyD88 and disruption in MyD88 and TLR2 disrupts signaling cascades generated by TLR1 and TLR6 [27]. During infection with *Plasmodium berghei* ANKA strain (a model for cerebral malaria), *Tlr2*−/− and *Myd88*−/− mice have been shown by some groups to be protected from development of cerebral malaria, although the effects of TLR2 have not been consistently observed and may depend on the genetic background of the mice [28–31]. Infection with *Plasmodium yoelii*, a model of acute uncomplicated malaria, had either a strong phenotype with impaired survival in *Tlr2*−/−, and *Myd88*−/− mice or no observable phenotypic difference [32, 33]. It is likely that there are multiple innate mechanisms that contribute to the host response against malaria infection, and single knockout inbred mice may not recapitulate the complexity of human disease.

In an effort to clarify the role of TLRs in human malaria, several studies have used genetic approaches attempting to detect associations between common genetic variants in the genes for TLRs and clinical outcomes in patients with malaria. In a group of malaria cases from northern Thailand with confirmed *P. falciparum* infection, none of the genetic variants tested were significantly associated with development of complicated malaria (Table 3), whereas increasing copy numbers of the major T allele of *TLR1* rs5743551 demonstrated a trend towards higher odds of complicated disease (p = 0.13). Notably, while this polymorphism is in high linkage disequilibrium (LD) with *TLR1* rs5743551 in Caucasian subjects, it was determined that in the Southeast Asian subjects from Northern Thailand that *TLR1* rs5743618 is in very weak LD with *TLR1* rs5743551 and is found at very low frequency [14].

The finding that *TLR1* rs5743618 is associated with higher parasitaemia on day zero in an Asian population is similar to an investigation by a Brazilian group that demonstrated an association between the G allele of *TLR1* rs5743618 and increased risk of developing symptomatic malaria after infection with *Plasmodium* [33]. The Brazilian group did not genotype *TLR1* rs5743551 in their study, so direct comparisons between their study and the studied population cannot be made. However, taken together, these studies suggest that genetic variation in *TLR1* affects host susceptibility to malaria disease severity, while variation in *TLR6* is less likely to play a significant role in these processes. This association is further supported by a study in a population in Cameroon that reported a different SNP in *TLR1* (rs4833095) was also associated with increased parasite burden, whereas a SNP in *TLR6* was not associated with any clinical phenotype [34].

Stronger evidence that *TLR1* rs5743551 may influence host responses to malaria is provided by the finding of a significant association between the copy number of the T allele of this polymorphism and increased parasitaemia measured on day 0 of enrollment. Subjects carrying the TT genotype had a nearly two-fold higher parasitaemia than subjects carrying the CC genotype (Table 3). Segregated analysis by ethnicity showed that the strongest effect of the T (minor) allele was present in the subjects of Mon ethnicity. This finding differs from those of the Brazilian group who found no association between *TLR1* variation and parasitaemia. In contrast, the population in Cameroon demonstrated a similar association with *TLR1* polymorphism associated with differences in parasite load. Explanations for the differing findings may be due to the different *TLR1* variants tested but could also include marked differences in environment (Brazil versus Thailand), and underlying population genomic differences. Furthermore, in the Brazilian study a substantial minority of patients were infected with *P. vivax* (39 %), whereas the population in Cameroon was similar to the studied population in that it was a population uniformly infected with *P. falciparum*. Several groups have demonstrated different host response to the various *Plasmodium* species. For example, higher levels of IL-10 have been documented in humans subjects infected with *Plasmodium vivax* when compared to individuals infected with *P. falciparum* [31–33].

## Conclusions

The particular strengths of the present study include detailed clinical information on a population uniformly infected with PCR confirmed *P. falciparum*. The current study is the first to document an association of *TLR1* rs5743551 with a clinical outcome in an Asian population with malaria. TLR6 has never been strongly implicated in the host response to malaria and no association with TLR6 SNPs and clinical outcome in malaria were found.

These data demonstrate that the G allele of rs5743551 in *TLR1* is associated with the host control of parasitaemia in Asian patients with acute febrile malaria. This assertion is consistent with the demonstrated ability of malarial GPI to activate cells through TLR2/TLR1 heterodimers [4]. From an evolutionary standpoint, this suggests that G allele of *TLR1* rs5743551 may persist in higher frequencies in non-Caucasian populations because of a beneficial effect on host fitness in environments with high prevalence of malaria. This is the first plausible explanation for the relatively high frequency of an allele that is associated with worse outcomes in tuberculosis, sepsis, and leprosy [14–16]. While parasitaemia is generally correlated with complicated disease, high parasitaemia alone is neither necessary nor sufficient to account for poor patient outcomes and other host factors likely influence clinical outcomes [24]. These findings stress the complex interplay between the human genome and endemic pathogens.
